# An Artificial Intelligence-Based Collaboration Approach in Industrial IoT Manufacturing: Key Concepts, Architectural Extensions and Potential Applications

**DOI:** 10.3390/s20195480

**Published:** 2020-09-24

**Authors:** Panagiotis Trakadas, Pieter Simoens, Panagiotis Gkonis, Lambros Sarakis, Angelos Angelopoulos, Alfonso P. Ramallo-González, Antonio Skarmeta, Christos Trochoutsos, Daniel Calvο, Tomas Pariente, Keshav Chintamani, Izaskun Fernandez, Aitor Arnaiz Irigaray, Josiane Xavier Parreira, Pierluigi Petrali, Nelly Leligou, Panagiotis Karkazis

**Affiliations:** 1 General Department, National and Kapodistrian University of Athens, Sterea Ellada, 34400 Dirfies Messapies, Greece; ptrakadas@uoa.gr (P.T.); lsarakis@uoa.gr (L.S.); a.angelopoulos@uoa.gr (A.A.); 2 Department of Information Technology/Internet Technology and Data Science Lab, Ghent University-Imec, Technologiepark 126, B-9052 Gent, Belgium; pieter.simoens@ugent.be; 3 Faculty of Computer Science, Department of Information and Communication Engineering, University of Murcia, 30003 Murcia, Spain; alfonsop.ramallo@um.es; 4 Odin Solutions (OdinS), 30820 Alcantarilla, Murcia, Spain; skarmeta@odins.es; 5 Pressious Arvanitidis, Kifissias Avenue 304, 152 32 Chalandri, Athens, Greece; chtrox@pressious.com; 6 Atos Spain S.A., Research and Innovation Department, Albarracín 25, 28037 Madrid, Spain; daniel.calvo@atos.net (D.C.); tomas.parientelobo@atos.net (T.P.); 7 Tractonomy Robotics, 8500 Kortrijk, Belgium; keshav@tractonomy.com; 8 TEKNIKER, Basque Research and Technology Alliance (BRTA), Iñaki Goenaga 5, 20600 Eibar, Spain; izaskun.fernandez@tekniker.es (I.F.); aitor.arnaiz@tekniker.es (A.A.I.); 9 Siemens AG Austria, Siemensstraße 90, 1210 Wien, Austria; josiane.parreira@siemens.com; 10 Whirlpool, Benton Harbor, MI 49022, USA; pierluigi_petrali@whirlpool.com; 11 Department of Industrial Design and Production Engineering, School of Engineering, University of West Attica, 12244 Athens, Greece; e.leligkou@uniwa.gr; 12 Department of Informatics and Computer Engineering, School of Engineering, University of West Attica, 12243 Athens, Greece; p.karkazis@uniwa.gr

**Keywords:** industry 4.0, artificial intelligence, IoT manufacturing, smart sensing, sensing-based IIoT

## Abstract

The digitization of manufacturing industry has led to leaner and more efficient production, under the Industry 4.0 concept. Nowadays, datasets collected from shop floor assets and information technology (IT) systems are used in data-driven analytics efforts to support more informed business intelligence decisions. However, these results are currently only used in isolated and dispersed parts of the production process. At the same time, full integration of artificial intelligence (AI) in all parts of manufacturing systems is currently lacking. In this context, the goal of this manuscript is to present a more holistic integration of AI by promoting collaboration. To this end, collaboration is understood as a multi-dimensional conceptual term that covers all important enablers for AI adoption in manufacturing contexts and is promoted in terms of business intelligence optimization, human-in-the-loop and secure federation across manufacturing sites. To address these challenges, the proposed architectural approach builds on three technical pillars: (1) components that extend the functionality of the existing layers in the Reference Architectural Model for Industry 4.0; (2) definition of new layers for collaboration by means of human-in-the-loop and federation; (3) security concerns with AI-powered mechanisms. In addition, system implementation aspects are discussed and potential applications in industrial environments, as well as business impacts, are presented.

## 1. Introduction

The adoption of innovative digital technologies, also referred to as Industry 4.0, is progressively leading to improved product quality, work safety, fault predictions and efficiency in energy use and production [1,2,3]. Industry 4.0 concepts are expected to significantly increase their footprint in industrial sectors by 20% in the next five years, since they allow leaner and more efficient production [4,5]. In this context, many manufacturing companies are interested in accelerating the adoption and integration of secure, trustworthy artificial intelligence (AI) [6]. In particular, AI-based manufacturing has the potential to improve the business key performance indicators (KPIs) of manufacturing processes by leveraging heterogeneous industrial big data analysis, information modelling and federation [7,8,9]. In this context, interconnection of AI-based manufacturing processes with currently deployed wireless networks is a challenging research field, especially when central processing is performed outside industrial premises [10,11]. However, most AI techniques are based on mathematical models that are difficult to understand by the general public, so most people use AI-based technology as a black box that they eventually start to trust based on their personal experience. The application of human-centric AI (HAI) in internet of things (IoT) systems, so that IoT systems cannot only learn from users but also provide easy-to-understand explanations about decisions or estimations is a new research field [12].

The industrial internet of things (IIoT) is a physical network of things, objects or devices (that contain embedded technology) for sensing and remote control, in an industrial context, that allows greater integration between physical and cyber worlds [13]. In the fifth-generation (5G) era, efficient, reliable and high-performance applications’ provision has to be combined with exploitation of capabilities offered by 5G networks. Optimal usage of the available resources has to be realized, while guaranteeing strict Quality of Service (QoS) requirements such as high data rates, ultra-low latency and jitter [14,15]. In this context, AI is critical to the cybersecurity aspect of an IIoT-enabled connected manufacturing environment, for accurately detecting and mitigating threats [16,17,18,19]. At the same time, introducing AI will lead to a more productive and safer working space, relieving human workers from routine procedures and employing intelligent machines and robots to perform heavy tasks, thus allowing human workers to focus on creativity, reasoning and decision making [20,21].

However, the adoption and integration of AI-based innovation in the manufacturing domain comes with a few hurdles and caveats that must be properly addressed in order to take advantage of its full potential, without jeopardizing the irreplaceable role of humans and the protection of sensitive data and procedures [22,23]. In this context, the goal of the study presented in this manuscript is to analyze an AI-based collaboration approach in industrial IoT manufacturing. To this end, architectural extensions to the Reference Architectural Model for Industry (RAMI) 4.0 approach are described, along with potential applications and business impacts.

The rest of the manuscript is organized as follows. In Section 2, the motivation behind our work is presented, while the overall contribution is described in Section 3. Section 4 describes in detail the proposed architecture, while implementation aspects are discussed in Section 5. Potential industrial applications are outlined in Section 6 while business exploitation opportunities are discussed in Section 7. Finally, concluding remarks are provided in Section 8.

## 2. Motivation

The motivation behind this work is the need to provide an IIoT-based system that increases performance and safety in the manufacturing domain. This system, which enables the digitization of the relevant processes in critical infrastructures like manufacturing plants, should be secure, reliable, resilient and privacy-preserving. At the same time, it should facilitate the interaction between humans and machines and support the collaboration between peer systems. In this context, AI has the potential to support several of these requirements in an efficient manner and, thus, it can be embedded into the fabric of a trustworthy IIoT platform.

Today, AI solutions are implemented in dispersed and isolated components of manufacturing IT systems. In most cases, the successful application of AI consists of pilots or testbeds to optimize specific processes (e.g., usage of computer vision for intelligent defect inspection). To realize the full innovation potential of AI, it must be regarded as a cross-cutting concern for manufacturing IT systems. Current Industry 4.0 reference architectures do not properly integrate the needed building blocks such as new deployment paradigms (e.g., edge-based learning to reduce bandwidth load on the enterprise network), scalable data-processing pipelines and information models, and AI-enabled digital twins used for monitoring and optimizing business intelligence [24,25,26]. In addition, the availability of big data has been one of the most important enablers for the recent wave of AI innovations [27]. Installation of sensors, the collection and curation of big data as well as modelling of all available information in an appropriate format incur significant infrastructure and labor costs. Moreover, every phase of AI algorithm design requires high-level skills (model selection, training, hyperparameter optimization). In the agile manufacturing of the future, these costs must be amortized over low-volume batches (even lot-size-one).

AI technologies should not only be used for data analytics in support of business intelligence, but also for automated decision making on manufacturing process parameters and configurations. Moreover, the rise of the “as-a-service” model means that on-site IT systems increasingly communicate with third-party cloud-hosted services. Unauthorized and malicious access to these systems may have not only a significant economic impact but also may compromise the physical integrity of human workers on the shop floor. Faulty behavior may also be an unintended result of wrong design, because AI algorithms are often black-box models (e.g., deep learning), while the inner workings of an algorithm fetched from a remote repository are not fully understood or the decisions of one algorithm create a conflict with other algorithmic decisions. Proper secure federation mechanisms and AI-based cyberattack risk analysis are crucial cross-cutting concerns in AI-based manufacturing systems. In addition, the performance of modern AI techniques requires large volumes of high-quality data which are often not available inside a single enterprise. From a business perspective, sharing data with other economic actors or even between different facilities of the same organization is often not feasible for reasons of Intellectual Property Rights (IPR) protection, General Data Protection Regulation (GDPR), cybersecurity, etc.

Finally, in recent years, machines in the manufacturing sector have become specialized agents that autonomously perform a given task at an outstanding quality standard with a high level of repeatability. Industry 4.0 will make machines increasingly smarter by using AI models [28]. In the next wave of innovation, sometimes referred to as Industry 5.0, AI techniques will be used to extend and improve the levels of communication and collaboration between computer systems and human workers.

With the objective to leverage unique human intellectual capabilities and to confirm and reinforce the role of human workers in an increasingly digitalized and automated environment, new intelligent design and decision-making tools must be developed to promote human agency and oversight, simplifying the understanding and usage of AI results and considering multiple collaboration schemes depending on the situation. Human-AI will work in tandem in any phase of the product construction process, from design, over intelligent manufacturing execution monitoring to predictive maintenance.

## 3. Contribution

**Figure 1:**
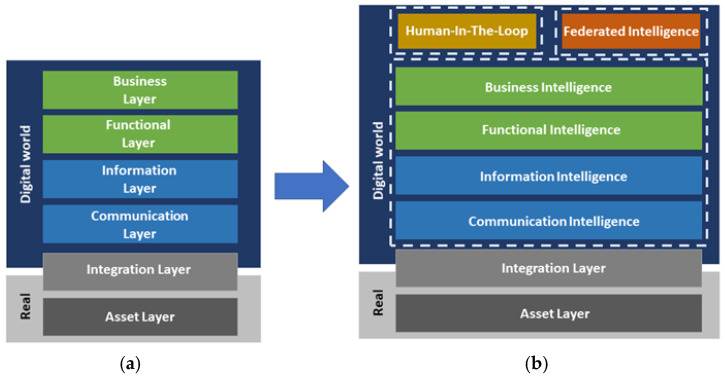
Reference Architectural Model for Industry (RAMI) 4.0 architecture (**a**) and proposed extensions (**b**).

The RAMI 4.0 (ZVEI German Electrical and Electronic Manufacturers Association, Frankfurt, Germany) provides a blueprint of manufacturing systems and has been developed to create a common perspective and develop a common understanding between all the actors involved. Structured along with well-defined layers (Asset, Integration, Communication, Information, Functional, and Business layers), RAMI 4.0 provides a service-oriented reference architecture that spans all elements and information technology (IT) components (from product up to the connected world) along the whole product life cycle (from design to maintenance). To address the aforementioned challenges related to AI introduction in smart manufacturing, we propose three types of extension to the RAMI 4.0 architecture, as depicted in Figure 1.

First, enhancement of all existing RAMI 4.0 layers, reflecting that AI is a cross-cutting concern affecting all functionality in IT manufacturing systems. Indeed, AI-augmented manufacturing requires novel forms of data processing and information modelling and AI will allow for more autonomous and automated business intelligence. Second, we define a human-in-the-loop layer that provides methods, models and tools to facilitate the collaboration among human and AI-based virtual entities within a manufacturing site, thus assisting humans to take the optimal decisions. Third, a federation layer is defined that promotes the exchange of knowledge in terms of training models, AI algorithms, threat analysis results, deployment recipes and best practices across manufacturing sites by integrating novel concepts, such as secure federated learning and AI-on-demand schemes.

Finally, one of the main challenges towards the implementation and adoption of AI concepts in IT manufacturing systems is the increased need for interoperability at different levels of the manufacturing ecosystem [29]. In this context, the application of existing standards for service-oriented industrial communication infrastructure by Open Platform Communications United Architecture (OPC-UA) and for semantic data exchange by Automation Markup Language (AutomationML) provides existing means for realizing the required connectivity and interoperability for intelligent co-operation in smart factories [30]. Therefore, integration and reuse with different existing and emerging solutions is of utmost importance.

In this context, our architectural approach relies on cloud-native tools for timely data collection, processing and curation, relying on the dynamic instantiation of data pipelines, while addressing security, privacy and confidentiality concerns across the physical and virtual entities. In this way, our approach transforms the collected data and information models into AI-enabled functional intelligence, leading to business knowledge, actionable insights and informed decisions, while being capable of recognizing complex events and process deviations that cannot be easily and timely captured with human judgment. Also, this ensures that our system is scalable to support manufacturers, ranging from Small or Medium Enterprises (SMEs) with limited on-premises resources, up to large industries with private clouds.

## 4. Description of Proposed Architecture and Main Components Functionality

**Figure 2:**
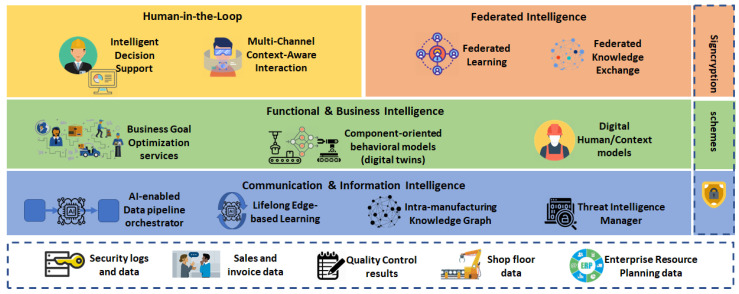
A high-level view of the proposed architecture.

In compliance with the RAMI 4.0, our proposed service-oriented architecture consists of the layers depicted in Figure 2, each one serving different functionalities and purpose, jointly fulfilling the requirements of the collaborative and intelligent factory of the future.

Besides extensions to the existing Business, Functional, Information and Communication Layers, the architecture contains additional layers for hardware-in-the-loop (HITL) collaboration and for collaboration through federation with other manufacturing sites, while security is a cross-cutting concern. In the following, we describe the functionality of each component as shown in Figure 2, along with its innovation perspective. Aspects regarding the implementation of the key AI-based components of the architecture are discussed in Section 5.

### 4.1. Communication and Information Intelligence

The main responsibility of the Communication and Information Intelligence layers is to perform all the needed actions on the factory-wide datasets so that the components of the upper layers can make decisions based on the outcome of AI algorithms running on top of such processed data streams or batch datasets. Raw (non-labeled) data generated by manufacturing devices are processed along with all other relevant info (e.g., data logs, quality control results data, etc.,) of the lower layer and properly transformed before passing to the upper layers of our architectural approach. The goal is to have an enriched metadata library covering various and diverse data creating during manufacturing procedure.

This layer also contains core functionality to deploy AI algorithms closer to the sensor (edge computing) and to detect shifts in the dataset statistics, indicating a need to retrain algorithms. In support of the two major categories of AI algorithms (data-driven machine learning [31] and knowledge-based models [32,33]), this layer contains both functionalities related to data logistics and shaping as to information modelling. Lastly, a threat analysis component constantly monitors all data flows running between services. The functionality of each one of the main components included in this layer is explained below.

The AI-enabled data pipelines orchestrator component enables the creation and deployment of data processing pipelines, with two major objectives: (i) the component should allow the possibility to set up pipelines consisting of typical data processing tasks (feature conversion, feature reduction, data anonymization and fusion, data cleaning, labeling and annotation, etc.) and AI models (used in the services of the upper layers). From this perspective, the component provides the mechanisms to create these pipelines easily as well as a set of out-of-the-box and extensible data processors to make the data handling easier and effective for the most common data used in the factory; (ii) the component should allow the deployment of the pipelines and the orchestration of the different components and frameworks used, including the AI models developed and made available via containers. To do so, the component should be able to automate the deployment process on distributed infrastructure (edge device, edge cloud, public cloud) and to orchestrate the different modules and exact frameworks needed to run the processes.

The lifelong edge-based learning component provides support for building deep-learning models that must be deployed on edge devices as part of data-processing pipelines [34,35]. Edge-based learning is required for latency-sensitive situations and/or when upstream bandwidth is insufficient, e.g., audio and video from an augmented reality (AR) headset or processing light detection and ranging (LIDAR) data on a mobile robot. The component will support novel neural network architectures that can be trained without requiring large amounts of labelled data and that are resource-efficient [36]. Moreover, as future factories will be constantly changing environments, the component will support AI algorithms that are not trained once on a single large batch of data, but that are frequently re-trained while being in operation.

The intra-manufacturing knowledge graph serves as the central point for knowledge management in the platform. This module combines and extends currently available models, both domain-specific and domain-independent, to enable knowledge representation and linkage. Moreover, it applies and extends state-of-the-art reasoning and graph analytics algorithms for link prediction and entity consolidation to discover relationships across the information coming from the different layers and modules.

The threat intelligence manager takes advantage of the collected and curated datasets and applies AI algorithms for executing threat analysis in order not only to predict potential cybersecurity incidents but most importantly to manage and mitigate such incidents in a timely manner. This component provides a solution that addresses the shortcomings of current signature-based methods (that can efficiently detect existing cyber-attacks, but are inherently incapable of discovering zero-day attacks where there is no predefined rule) and anomaly-based methods (that can detect known and zero-day attacks with some limitations of false-positive rates, but cannot detect attack types such as distributed denial of service).

### 4.2. Functional and Business Intelligence

This layer accommodates all services and components modeling the status and behavior of all functions and assets (including humans) of the manufacturing process. These models are built using AI algorithms and trained models, leveraging the data processing capabilities and information models from the lower layers. On top of these, other AI-enhanced services will be built, either for automated business goal optimization or in processes with HITL. In the following, the components belonging to this layer are presented.

The Component-oriented behavior models are digital twins with behavioral models of individual components; in this way, they are moving away from system-wide behavioral models currently used. Each component not only digitally represents the state of assets and properties through the asset administration shell (thus providing digital twin) but also models the state transitions, e.g., in the form of a finite state machine. This allows us to mirror the state of the production process in digital twins, including the logic that determines the transition to other production steps or states. Learning behavior on a component basis and forming a complete model from different components has the advantage that if one component is replaced, only the associated model must be re-trained without affecting the system-wide behavior model. This reduces the time it takes to adjust the complete behavior model if a component is exchanged. Another advantage of this approach is that component-based models are likely more reusable in other contexts, enabling collaborative learning and monetization of components.

The digital human/context models have as main objective to achieve efficient human-centric intelligent control systems by learning and modelling human workflows, strategies and decisions as part of cooperative tasks. Environment and context parameters are integrated with the resulting operational models in order to address complex situations that are characterized by different levels of uncertainty. Two main types of model are developed: classification models that are able to recognize situations and models to predict human actions and decisions in a workflow (e.g., short-term future movements of human operators in the shop-floor considering current situation, typical decisions for a given event). In this way, more intuitive user interfaces are developed, since the AI system knows how the human most likely will interact while keeping the human in control. This layer also contains services for business goal optimization, which are application specific. Potential applications of the platform are discussed in Section 6.

### 4.3. Human in the Loop

This layer provides innovative tools that will facilitate intuitive and efficient collaboration between humans, machines and AI systems allowing them to take advantage of each other’s strengths for more effective cooperative and intuitive task execution and decision making.

The multichannel and context-aware interaction manager creates innovation by moving beyond traditional interaction mechanisms between humans and IT systems on the shop floor, such as command-line computer screen, pendant consoles and buttons. A good user interface is intuitive and does not require human operators to be educated with specific structures or behaviors. Voice commands could be such a solution, but due to the noise from machines voice is difficult to catch and process by AI systems on a shop floor. Instead, this component allows for multiple simultaneous input channels (voice, gestures, facial expressions) that provide redundancy in the input, thus overcoming the decreased robustness of individual channels, e.g., due to variable lighting or noise on the production floor. To further improve the spontaneity of the human-machine interaction, context information is taken into account, such as the location of the operator or current production parameters [37].

The intelligent decision support system (IDSS) will allow humans to consider extensive experience, expert knowledge, context information and empirical data to make rational decisions at the strategic or business level, e.g., to maximize the performance of the manufacturing system. In our proposed approach, traditional decision support systems (DSSs) which are widely used in the manufacturing domain will be extended with novel capabilities, e.g., digital twins or threat intelligence models. The integration of digital twins and IDSSs has great potential: the former do not have knowledge about enterprise business goals and constraints, while the latter require holistic and advanced simulation models to give recommendations. Thus, advanced data analysis techniques will make possible to rely on objective and evidence-based insights.

### 4.4. Federated Intelligence

The Federated Learning component aims at solving the problem of data collection for feeding or training AI models, while assuring the ownership and confidentiality of the data. In manufacturing, most (if not all) data and information are confidential because they relate directly to details of the production process, product characteristics, volumes, etc. By applying private set intersection (PSI) technologies [38], this component enables two parties holding a set of private information to identify the intersection of their information sets without revealing any information except for the intersection, while technologies like the open-source framework TensorFlow Federated provide support for decentralized AI models learning or computation over locally controlled data sources [39].

The inter-manufacturing knowledge exchange serves as an interface for knowledge exchange across manufacturing sites or distinct manufacturing processes. Since there is a need to control what information is exposed and exchanged, rather than allowing open access to the local knowledge repository, this component contains a query engine to handle external requests. Such query engines also enable the realization of a federated query-processing mechanism over multiple sites.

### 4.5. Security and Authorization

Our proposed platform also addresses security and authorization requirements on information and data sharing. Towards this end, the signcryption schemes component provides an effective and scalable solution, based on new cryptographic primitives, such as ciphertext policy attribute-based encryption (CP-ABE) schemes. The scheme will encrypt the data according to an access control policy based on a list of attributes and guarantees that the user’s keys are associated with the user’s descriptive attributes. Therefore, the data owner can freely define the access control policies that are used to encrypt the information, being assured that a user can decrypt the information if and only if their secret key matches the access policy used to encrypt the data. Thus, adopting this novel approach [40], this scheme heavily reduces the administrative effort for key sharing and management, while ensuring end-to-end information protection. To take into account needs, in terms of processing resources and time to encryption, which are quite usual when dealing with shop floor devices, CP-ABE can be combined with symmetric encryption schemes (e.g., Advanced Encryption Standard, AES) ensuring the required trade-off between granular information protection and performance.

### 4.6. Cybersecurity for Artificial Intelligence (AI)

The presented framework incorporates AI in several components of an IoT-based manufacturing system. However, this introduces further concerns regarding the security and trustworthiness of these AI systems themselves.

Artificial intelligence attacks, i.e., attacks on the AI algorithm, can take two forms: input attacks and poisoning attacks. The former consists in manipulating the input to the AI system during the operation phase so that it delivers the wrong results. Input attacks are relatively easy to launch and succeed since they do not require a manipulated AI system. Poisoning attacks, on the other hand, have to do with the corruption of the process used to build the AI model. In this case, inaccurate or mislabeled data are provided to the model during the training phase to manipulate the learning process. This type of attack can also be launched against federated learning; in this case, manipulated data or an algorithm of a member of the federation can result in the corruption of the global model.

The defense against adversarial attacks, which is the core element of trustworthy AI, has recently received much attention [41]. To protect AI systems, traditional cybersecurity mechanisms and policies can be used as a starting point. In this regard, the security-related components of the proposed architecture, which support confidentiality, integrity and threat detection, are able to guarantee a first level of protection. However, providing complete cybersecurity for AI, and especially doing so for the case of IIoT, requires further enhancements to address the unique vulnerabilities of these AI systems and it is left for future research.

## 5. Realization Aspects of AI-Based System Elements

In this section we discuss challenges regarding the implementation of the innovative AI-based components of the proposed system. Towards this end, relevant existing technologies and approaches are reviewed, limitations are identified, and new solution perspectives are presented.

### 5.1. AI-Driven Modelling of Manufacturing Assets

Digital twins are virtual, high-fidelity models of the current state and internal behavior of physical assets on the shop floor [25]. These digital counterparts are created either from knowledge engineering and expert modelling or using data-driven techniques with sensor data collected via industrial IoT technologies [42]. Modern production environments need to deal in an agile way with reconfigurations, e.g., due to low-volume production. Regardless of whether digital twins have been created in a model-driven or data-driven manner, the different processes occurring in different sectors are making the digital twins defined ad hoc, and there is a lack of information models and process libraries that allow users to replicate and scale their digital twins. Other current limitations today are that experts in simulations and modelling are not available in SMEs and large enterprises, and that data-driven digital twins are usually trained on data collected at system level. These limitations make it economically costly to develop novel digital twins, which is often needed in modern manufacturing with agile reconfigurations.

Data-driven digital twins are built using machine learning. In this domain, deep learning (DL) is the dominant branch today, yet DL comes with its own problems: it requires domain knowledge to select the appropriate machine learning pipeline and it is very resource hungry in terms of computing and storage resources [36,43,44]. AutoML approaches can be used to automate the pipeline building, but these tools are currently unable to deal with sequential data that is usually used in building behavioral models. For the IT resource aspect, processing all raw data on a public cloud infrastructure is an unscalable solution for many manufacturing companies, either because there is too much sensor data to upload, the latency to the cloud is prohibitive or because the sensor data is too sensitive and the company does not want to expose this. Therefore, edge computing has been proposed and several reference architectures for edge computing in Industry 4.0 have been proposed [26], such as the far edge reference architecture that includes blockchain support (http://far-edge.eu). The term edge computing covers various deployment options: from an on-premises datacenter to embedded computing, e.g., on AR glasses or on mobile robots. Techniques to make deep neural networks available for the latter form of edge computing start to find their way into production as more user-friendly tools become available. For instance, TensorFlow Lite allows converting a trained model for deployment on microcontrollers or embedded Graphics Processor Units (GPUs).

For the modelling of manufacturing assets, we propose a solution based on semi-automated construction of digital twins from components. This solution combines black-box data analytics (deep learning, also on edge devices [45]) with grey-box models (e.g., finite automata) to model the state and state transitions of individual components. The creation of models of the state and behavior can be done in a semi-automatic manner, using a newly devised AutoML tool that takes as input vector representations of sequential input data [46]. To link these component models, it is necessary to capture all production and process information in a knowledge graph [47], which constitutes an information model optimized for relationships and allows to extract novel knowledge by means of reasoning and graph analytics.

### 5.2. Multi-Channel, Context-Aware Interaction on the Shop Floor

Research on verbal-based human-AI communication has recently seen large advances. Modular dialog systems consist of, at least, (i) a natural language understanding (NLU) module; (ii) a dialogue management module; and (iii) a knowledge base. In order to extract information from human utterances and to track dialogue states, rules and machine- and deep-learning techniques have been used. So as to store knowledge, besides traditional databases, modern solutions make use of semantic web technologies to provide semantic processing of commands. As an alternative to modular systems, end-to-end approaches using deep-learning techniques are widely considered.

The latest developments [48,49] allow humans to convey information with AI systems through multiple channels by integrating advanced human-machine interfaces (gestures, facial expressions). These interfaces, which can be obtained by using 2-D and/or 3-D cameras and other sensors, such as gyroscopes or accelerometers, offer information related to the context and the situation that is relevant to the interaction.

For voice understanding, it is necessary to have a fair amount of data. Since the investigations in industrial settings are at an early stage, the data available for training the systems are scarce. To cope with such a problem, a promising solution is to develop a rule-based system as a first step to perform NLU and generate data for future training of supervised algorithms. In this approach, the system will automatically learn from new sequences while preserving previous knowledge. In order to store knowledge, semantic web technologies can be used [50,51]. These technologies, besides acting as a database, enable the system to reason and make inferences from human commands, emulating human reasoning. Moreover, semantic web technologies allow the interoperability between other knowledge bases that capture context information (such as the knowledge graphs).

### 5.3. Intelligent Decision Support

Integrating traditional DSS with AI can increase the efficiency in the decision-making process and introduce higher levels of automation, resulting in the appearance of problem-oriented intelligent DSS (IDSS) or knowledge-driven DSS. Most of the ongoing efforts in this sense are still limited to the application of rule-based expert systems, or machine-learning or genetic algorithms [52] on raw data [53]. One of the problems that affect DSSs is the need to rely on inputs collected, modelled and generated by human operators. Moreover, current approaches fail to learn from decisions taken by humans.

Regarding the realization of this component, we propose a hybrid approach, integrating knowledge bases (the behavioral models and the knowledge graphs) with data-driven methods. In particular, the use of advanced deep-learning models enable knowledge to be automatically synthesized from the raw multidimensional data collected from the entire manufacturing organization. The IDSS may also include continuous learning functionalities in order to increase its capabilities in time, extracting knowledge from the decisions taken by the human operators. This information can be used to provide better recommendations and to adapt behavior to changing conditions.

### 5.4. Threat Intelligence Manager (TIM)

Conventional security methods are not sufficient as industrial networks are foreseen to be frequently upgraded and their topologies are subject to changes. In this direction, adopting AI-based approaches is indispensable, in order to significantly enhance the network intelligence and security of industrial processes [54]. Towards this end, the AI algorithms can identify small anomalies in data flows from various sensors, which are the source of industrial data, thus constituting an important enhancement of the cybersecurity within factory halls. Also, AI enables the detection of data integrity threats, relying on training with legitimate traffic in order to learn the regular sensed data flow in the network. In the case of malicious activity, various AI algorithms, such as naïve Bayes, random forests and support vector machine (SVM) have been proposed [55]. Besides the flow characteristics, AI can detect data sources with abnormal behavior, unjustified excessive data traffic or stops to regular operational traffic. AI algorithms in this field are mainly based on SVM and deep belief networks [56]. Unsupervised AI techniques, as well as k-nearest neighbors (KNN) have been used against authentication attacks, such as man-in-the-middle, offering improved resistance in such scenarios.

By exploiting advanced AI methods, the threat intelligence manager (TIM) component intends to model the dynamic interactions of Industry 4.0 subsystems and discover known and unknown attacks, while surpassing existing signature- and anomaly-based methods. This will provide an efficient solution for the detection, prediction and timely management and mitigation of cybersecurity incidents and abnormal traffic activity. The realization of this component can be based on the Beta mixture and hidden Markov models (BMM and HMM), aiming at accurately and effectively detecting and discriminating normal and attack data [57]. In HMM, the first component, namely the BMM will fit multivariate time series of physical and network data and will stand for the input of the second component, i.e., the HMM. The performance of the HMM will be enhanced by excluding irrelevant features and reducing sensor and network dimensionality, through an independent component analysis (ICA) technique [58].

### 5.5. Federated AI across Manufacturing Sites

The proposed system considers both the federation of knowledge using distributed querying, and of federated learning to avoid sharing raw data. In contrast to knowledge graphs such as Wikidata (https://www.wikidata.org), which model general data, intra-organizational corporate/enterprise knowledge graphs model data relevant only to the organization itself, e.g., products or processes. It many practical scenarios, information is distributed across various sites, instead of being kept in a centralized location. This indicates the absence of a unique endpoint for data retrieval. In this context, federated query processing [59] is an active research field dealing with techniques for proper delegation of the execution of parts of queries to specific sources. Current federation engines support around the order of 10 sources, which is insufficient for highly distributed and decentralized environments such as the Industry 4.0 ecosystem. In such an environment federated queries over 100 to 1000 sources will be required, for example when integrating knowledge graphs across multiple manufacturing sites. To improve the scalability of such federations, aggregation techniques could be used, where one or more independent aggregators would continuously crawl sources, and maintain data summaries [60].

In many domains, including manufacturing, a single party does not have sufficiently large data sets for training AI models. Joining datasets held by different actors can address this issue. Often this cannot be performed due to data being confidential (e.g., production data, Bill of Materials—BoMs or normative constraints). Indeed, usually manufacturers do not want their data to leave their premises and be disclosed to cloud providers or third-party organizations. To this end, federated learning provides a solution enabling machine learning over distributed and decentralized datasets. Currently, federated learning is being adopted in different scenarios such as banking [61] and healthcare [62].

A related approach is represented by private set intersection (PSI) that addresses the issue where different actors have information, often complementary, about some common business, but they cannot or do not want to share. To this end, PSI represents a solution that enables two parties holding a set of private information to identify the intersection of their datasets without revealing any other information except for the intersection itself. Some PSI protocol offers the possibility to perform simple operations on the intersection of the data sets [63]. There are currently many research activities to develop faster or more resource efficient PSI protocols [38,64]. In this respect, federated learning opens up new business models (AI as a Service, AIaaS) to analyze data provided by a customer. However, on the one hand the service provider will never provide the AI model to the customer (as it represents its most valuable asset), and on the other hand the customer may not be open to provide its data to be analyzed by the service provider or to refine the model. As is evident, federated learning represents a solution to this problem enabling both the service provider and its customer to achieve their objectives while preserving the business assets.

Regarding the query processing across sites, an efficient solution could be obtained by creating data summaries that are suitable for the underlying data representation and by extending the existing query engines to make use of such summaries for efficient federation.

### 5.6. Further Implementation Considerations

Several of the components of the proposed system have been partly tested in research and innovation projects during the last few years. This, in turn, illustrates their validity as separate parts and puts closer the solution described in this paper.

In this context, an indicative example is the IoTEP, the Internet of Things Energy Platform [65]. This solution was implemented as part of the European project ENTROPY [66], which investigated the use of information and communication technologies (ICT) to promote energy saving behaviors in buildings. The IoTEP had an analytics support layer (ASL) and a services layer, in which a series of algorithms performed advanced analysis of the data in order to provide added value for the user. Within the ASL there exist the virtual energy-building areas generator (VEBAG), which is an example of a digital counterpart of the building understood as an operational space. In the same way in which the areas were considered as building blocks of the entire building, in the solution shown in this paper, the different manufacturing areas can be considered as building blocks of the complete process. Once this is done, it is possible to generate in an automatic manner intelligent services in parts of the building blocks or in the process as a whole.

One of the challenges to perform this in industry is the handling and labeling of data of very different types. In IoTEP, (transferable to this case) a built-in Representational State Transfer-based (RESTful) capability was created as aggregation functions. These features were included thanks to Comet (a short-time historic (STH) component of the FIWARE project ecosystem [67]) that performed this in the homogenization layer.

Another example of the application of intelligent modules as the one suggested here, is in a solution for intelligent agriculture [68]. In this case, more weight was given to edge computing to perform analytics tasks due to the nature of the smart agriculture. In this case, a platform based on FIWARE is connected via a context broker to facilitate the annotation of the data, which through Cygnus (a connector in charge of persisting context data sources into other third-party databases and storage systems) connects to the big data solution (Hadoop Distribute File Systems, HDFS), and through standardized Next Generation Service Interfaces (NGSIs) to Comet. Thanks to this data cloud configuration, the users can enjoy intelligent services via a REST Application Programming Interface (API).

## 6. Potential Industrial Applications

A platform based on the proposed architecture can be used for enhancing the performance of a variety of operations in the manufacturing domain. In the following we describe two indicative industrial application scenarios where different system components can be exploited for optimizing manufacturing logistics processes, and facilitating zero-defect manufacturing. For both scenarios we assume a realistic current practice and demonstrate what advancements the proposed system may bring to the relevant case.

### 6.1. Secure Autonomous Mobile Robots (AMR) Fleet Management for Time-Guaranteed Delivery of Goods at Assembly Lines

Many manufacturing companies are currently investing in autonomous mobile robots (AMRs) for supporting logistics operations regarding the transportation of goods from warehouse to working cells along the assembly lines or from the assembly line to dedicated areas for quality check purposes. Compared with automated guided vehicles (AGVs) that can only move along marked lines or wires in the floor, AMRs move flexibly in a factory, recognizing and avoiding obstacles at high safety levels and are easily reprogrammable. AMR-based deliveries are ideal for several types of factory because rigid automation like conveyors can be avoided, the system can work around people and machines at high levels of safety and reduce labor needs. In addition, accident risks are reduced by avoiding heavy vehicles like forklifts or tuggers in fast-moving intralogistics areas during peak activities. AMRs need to trade-off operational efficiency (uptime, speed, accuracy) with safety while achieving business objectives. Simultaneously, AMR downtime is undesirable and should be reduced as much as possible.

**Figure 3:**
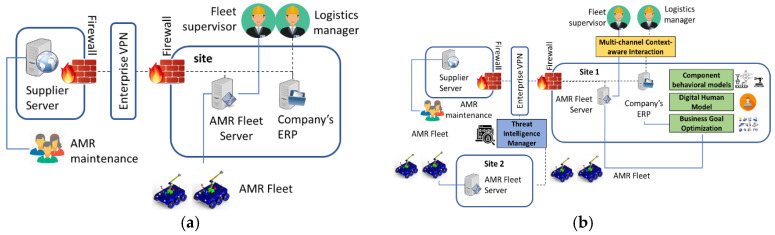
Current (**a**) and system-enhanced (**b**) autonomous mobile robots (AMR) production logistics.

This scenario assumes that a company has implemented a fleet of AMRs connected to a network infrastructure that allows for monitoring by company administrators for business and safety, and by the AMR supplier for maintenance. The current AMR production logistics is supposed to be carried out by the system illustrated in Figure 3a. Furthermore, it is assumed that there are two issues that need to be addressed in an efficient manner: (a) unpredictable delivery times and downtime, and (b) vulnerability to network attacks.

The manufacturing company is looking for predictable, deterministic material deliveries by AMRs that scale with the factory’s configuration and activities without tedious (re)programming. The deployed AMRs operate on highly dynamic production floor environments with people and vehicles. The AMRs are mostly individual units that plan isolated trajectories, frequently just stopping and waiting for an obstacle to pass. This results in missed delivery deadlines and stalling of downstream work cells. In addition, AMRs are currently unable to recover from system errors and failures internal or external to the AMR, for example, while positioning at a packing station or driving with a worn wheel. This results in expensive delays and downtimes. The complexity increases with every new AMR added to the fleet. Lastly, to obtain information about material delivery and AMR status, workers must use a PC or start an app on a tablet, which interferes with their activity.

The AMRs need to be networked to the vendor- or enterprise-hosted AI cloud services to deliver real return on investments. Operationally, shop-floor supervisors and safety managers need situational awareness of the AMR fleet anywhere in the factory. AMR supplier companies may also want to offer remote maintenance services for their AMRs and thus need access to their customer networks. This opens opportunities for third-party attacks which can shut down production, or even compromise site safety. The solution must offer active threat-analysis that offers trustworthy insights that do not disrupt workflows through false positives or negatives. The fact of connecting the AMRs to the network infrastructure implies an inherent cybersecurity risk due to dynamic software vulnerabilities that affect the different software components and communication protocols. Indeed, the attack surface that could be exploited also implies safety risks for the human operators sharing space with the robots since an external attacker could gain control of them. Another particular risk arises since robots are equipped with many sensors and thus, they may leak confidential information about the shop floor. The manufacturing company needs the means to manage such risks. It also could cause big economic losses or reputation damage to the company if a serious accident is caused or if sensitive information is leaked.

*Advances leveraging the proposed platform*: the platform can provide a secure AI infrastructure and associated services to optimize the AMR fleet business goals of delivery time guarantees and preventive maintenance by learning from patterns of business process models and anomaly detection techniques, as illustrated in Figure 3b. At the AMR unit level, prediction of AMR downtime can be done using multivariate statistical models on data of key AMR system parameters, e.g., sharp drops in battery levels or recurring wheel slips. Edge-based (i.e., on-robot) learning can be used to reduce the amount of data uploaded via the customer network to AMR supplier’s cloud back-end and assure the confidentiality of production activity related data. The proposed platform can also run AI observers that collect data about typical movement patterns on the floor (e.g., edge-based learning on surveillance camera data) and about how fleet behaviors relate to production goals (e.g., from Enterprise Resource Planning—ERP and Manufacturing Execution System—MES systems). Also, data on other factors that may affect the delivery time can be analyzed, such as production plan, production calendar by department/line (shifts, breaks, etc.), type of products, etc. All this data can be used to develop an AI engine for fleet management that adjusts AMR unit parameters (speed up, slow down, recharge, change route, etc.) to achieve determinist time-constrained navigation goals under unexpected conditions, such as roadblocks or production target changes. Concretely, the algorithm can be trained in a simulator that takes as input digital human models, behavioral models of the AMR and other components.

Workers in this scenario can be equipped with a mixed reality (MR) application that presents live information about material delivery and that allows them to take corrective actions in case of problems. The AR/MR application can be available on wearable devices, being able to support gestures to reduce impacts on workers’ operativity. This component exploits features in the human-in-the-loop layer of the proposed system, concretely supporting interaction between people and AI.

As unauthorized access to machines and data might compromise the physical integrity of human workers, communication between AMR, the platform and AMR supplier’s cloud back-end can be secured by signcryption schemes provided by the system. The manufacturing company can benefit from the integration of an instance of threat intelligence manager that will apply AI methods in order to identify anomalous data patterns that may be indicative of an ongoing cyber-attack. Using the private set intersection technologies in the TIM, the company will be able to assess which information is exchanged to the cloud-backend of suppliers, without requiring access to the internals of third-party services. Secure federated learning can be used to exchange information and module updates with the threat intelligence manager deployed on the site of another company participating in the federation through interfaces to exchange information about cyber threats and attacks.

### 6.2. Zero-Defect Manufacturing (ZDM) and Cybersecurity for Web-to-Print (W2P) Service

This scenario involves a company in the printing and offset manufacturing sector that aims to reduce costs associated with defects in the manufacturing process and enhance the security of its IT infrastructure. The company offers W2P services to its customers, allowing customers to design, edit and approve digital content as well as check the status of digital or offset printing process via a web interface. The printing process is quite complex and often requires manual interventions from the personnel. Defects along the manufacturing process have a major impact on the company’s financial losses. Despite the efforts spent on the formalization and standardization of the different procedures across the departments as well as on providing incentives to the personnel to provide products of high-quality, defective products (books, leaflets, printed advertisement material, etc.) appear along the manufacturing process. Moreover, the acceptance of the final product is partially based on the customer’s subjective area.

**Figure 4:**
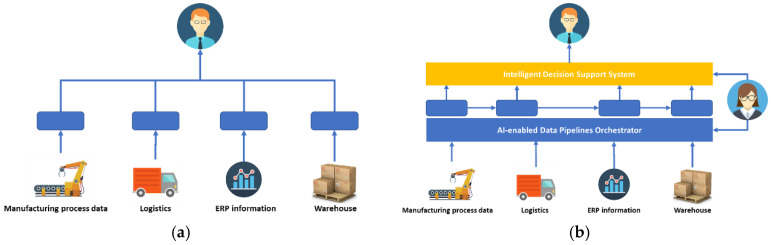
Current (**a**) and system-enhanced (**b**) ZDM solution.

The process of manufacturing in the company includes several departments (sales, invoice, purchase, offset printing, bookbinding, quality control, warehouse and delivery), running processes related to their specific tasks. In order to solve the defect manufacturing issue, the company stores and analyzes the deficiencies in the manufacturing process, thus holding a large dataset of production data (including ink level, printing speed of the machines, idle time between products, factory environmental conditions, results from color spectroscopy, mean time of the product remaining in the warehouse, scrap materials, etc.) along with all the defects that have occurred (time and frequency of occurrence, fault causes, percentage of wasted resources, estimated financial loss, etc.), an approach illustrated in Figure 4a.

The analysis of these datasets has led to some first improvements in product quality, but there is still significant space for further improvements and minimization of financial losses and product quality improvement.

The main reasons that create the problem and still remain unsolved are:

- The number and the diversity of characteristics of the orders do not allow for common process standardization.
- The data and information collected across the departments through manual and automatic procedures are solely processed by each department, without considering factory-wide optimization metrics.
- The deficiencies are observed, in the majority of the cases, during the quality control of the final product, thus leaving no space for corrective actions.
- The lack of personnel knowledge and training on the use of innovative tools that would automate the procedures (at least partially), letting personnel only make the final decisions.

**Figure 5:**
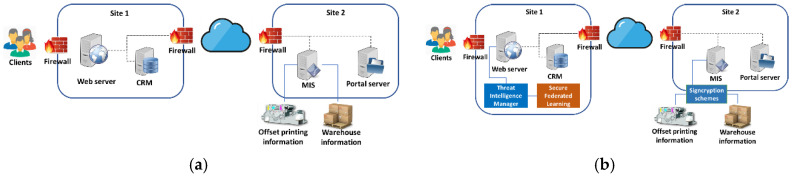
Current (**a**) and system-enhanced (**b**) web-to-print (W2P) solution.

At the same time, the company aims to reduce the cyber-attack surface of its IT infrastructure that is distributed in two sites. To create their printing file or obtain real-time information about the file printing status, clients connect to the W2P web service in the first site. Also, customer relationship management (CRM) is installed on this site. The servers in this site are connected to the management and information system (MIS) of the printing machines in the second site, in order to schedule the timing of the printing job according to the rest of the printing jobs in the factory. A high-level architecture is depicted in Figure 5a.

The connection to the second site of the company where the production line, warehouse and financial services are hosted, is achieved through two hardware firewalls with basic security services subscriptions enabled. On the second site, the portal server is used to store the uploaded files which will be used to the production line of the company, while the management and information system (MIS server) provides financial information, status of the printing process, and other types of information for the printing job status, such as warehouse information.

Since customers have access to a web server in the company network, and because the IT systems are physically distributed over two sites, there are many possible points of attack. Thus, W2P service offers an attack surface to cyber attackers to intrude the network and gain access to customers’ financial data and even control the printing machines, leading to the disruption of the company production line and even to potential safety risks for the human operators.

*Advances leveraging the proposed platform:* several elements of the proposed system can impact the business of such a company, as illustrated in Figure 4b. The dataset already collected by the company provides a head start to start building decision support algorithms. This dataset can be complemented with a knowledge graph-based model that captures all the parameters and configuration details of the individual production phases and links these together. The combination of data analytics and information modelling will allow for an intelligent decision support system that can be used for what-if analyses, projections, and root cause analyses. For scalable processing, an AI-orchestrated data pipeline can be set-up to collect all data from the departments and transform them into a semantically interpretable format using the knowledge-graph model. The data pipeline may cover both historical and real-time data.

Components of the proposed system can also be used in a principled approach to mitigate the security and data leak risks, as it can be seen in Figure 5b. First, by adopting novel signcryption schemes, manufacturing data exchanged between entities in the production line can be duly protected from authentication and confidentiality-related attacks from the internal network of the company. Secondly, the integration of the TIM can provide the means for the timely and effective counter measuring cyber threats and attacks. The TIM may build on knowledge and information on security attack signatures that are obtained via federated learning with another instance of the federation.

### 6.3. Testing and Validation

In testing and validation procedure, the goal is to define a set of KPIs per potential use case and validate the performance of our proposed AI-based approach via execution of the trials and validation. The evaluation plan includes technical, business and ethical validation metrics, since not only the technical requirements will be assessed but also the business feasibility and impact as well as the ethical issues related to the introduction of novel AI concepts and models.

## 7. Potential Business Impacts

The first and primary business impact of the proposed approach is on the manufacturing domain. Namely, through the proposed tools and components, manufacturers will be capable of realizing agile production processes and improve the quality of products and processes. This will help them to be more competitive in the market and thus increase their market share. Apart from this main business impact, additional business impacts can be identified as discussed below.

### 7.1. Enable New Digital Twin-Relevant Business Models and Revenue Creation Streams

The federated intelligence layer introduced in our approach enables new business models (e.g., high-quality AIaaS), as well as the collaboration of different industries towards the creation of digital twins. Any digital twin of a physical object captures and exhibits the behavior of the physical object to a certain accuracy and with respect to specific functionalities which may be of interest to different people inside or across different manufacturing plants. For digital twin value to be recognized [69], the insights need to tie directly to a workflow/task and accompanying goal. Thus, any digital twin of a specific object created by an industry can be traded and furthermore, additional data to improve its accuracy or to enrich the aspects captured by every digital twin is also possible. This implies that the data collected within any industry can be re-used and monetized. The definition, implementation and promotion of the federated intelligence layer can further fuel these interactions and make it possible to happen in real-time. Furthermore, our approach places emphasis on the collaboration between humans and AI which is a key aspect for the digital twin concept to be effectively used.

### 7.2. Foster the Competitiveness of Internet-of-Things (IoT) and AI Industries

The presented approach boosts the innovation potential of IT-, IoT- and AI-related companies through the definition of an architecture that facilitates the collaboration between manufacturing machinery, AI and humans with the already explained anticipated benefits. IT companies can offer open interfaces or adopt the presented interfaces so that their solutions are extendable to support novel collaborative AI applications. Last but not least, companies with expertise in AI for manufacturing can create significantly higher revenues by being capable of integrating their components with IoT and IT systems from different vendors/creators.

## 8. Conclusions

A novel architectural approach has been proposed, that extends the RAMI 4.0 architecture in the context of adopting AI systems in the manufacturing area. At the core of this approach is the provision of components for timely data collection, processing and curation, relying on the dynamic instantiation of data pipelines, while addressing security, privacy and confidentiality concerns across the physical and virtual entities. The collected data and information models are transformed into AI-enabled functional intelligence, leading to business knowledge, actionable insights and informed decisions, while being capable of recognizing complex events and process deviations that cannot be captured easily and in a timely way through human judgment. The provided functional intelligence is leveraged by components for secure communications, federation and exchange of data, knowledge, trained AI models or optimized AI algorithms across manufacturing sites and stakeholders, as well as by components that support efficient interactions between humans and AI systems through intuitive interfaces and facilitate informed and timely decisions regarding the operation of the manufacturing system.

The study of industrial applications regarding the optimization of the manufacturing logistics processes, and the facilitation of zero-defect manufacturing, illustrated the potential for practical applicability of the proposed architectural elements. Finally, the elaboration on the challenges regarding the implementation of the key AI-based components of the proposed system and the review of existing solutions identified areas where new approaches are needed to overcome current limitations and deliver effective and scalable solutions that can be exploited in new business models.
